# Winners of the Consortium of Universities for Global Health–Global Health: Science and Practice Annual Student Manuscript Contest

**DOI:** 10.9745/GHSP-D-17-00053

**Published:** 2017-03-24

**Authors:** James D Shelton, Pierre Buekens, Elizabeth Grant

**Affiliations:** a Editor-in-Chief, Global Health: Science and Practice Journal, Baltimore, MD, USA.; b Chair, Consortium of Universities for Global Health, Washington, DC, USA, and Tulane University School of Public Health and Tropical Medicine, New Orleans, LA, USA.; c Chair, Research Committee, Consortium of Universities for Global Health, Washington, DC, USA, and The University of Edinburgh, Edinburgh, UK.

## Abstract

The 2 inaugural winners of the CUGH–GHSP Annual Student Manuscript Contest describe (1) the American Mock World Health Organization model for engaging students in global health policy and diplomacy, and (2) a successful Indo-U.S. twinning model of global health academic partnership led by students.

See related articles by Lei and by Soni.

This issue marks the inauguration of publication of the first 2 winning articles from the Consortium of Universities for Global Health (CUGH)–Global Health: Science and Practice (GHSP) Annual Student Manuscript Contest. Each year at the CUGH annual meeting, we intend to select the best manuscripts from among student papers submitted for this competition. Manuscripts are judged by members of CUGH's Research Committee and one or more GHSP editors. Winning manuscripts are announced at the annual meeting and, assuming they undergo the further appropriate peer review, published in a subsequent GHSP issue.

The 2 winning articles in this issue are:

- Lei et al. American Mock World Health Organization: An Innovative Model for Student Engagement in Global Health Policy^1^
- Soni et al. RAHI–SATHI Indo-U.S. Collaboration: The Evolution of a Trainee-Led Twinning Model in Global Health Into a Multidisciplinary Collaborative Program^2^

The articles are especially notable for 2 reasons. First, they provide a healthy amount of rich process description, in keeping with GHSP's strong interest in documenting not only results but also how interventions are carried out. Thus, readers can better understand how implementation might be adapted for other situations. Second, both articles reflect a high level of student initiative and leadership in carrying out the activities described in the papers.

One of the major strengths of CUGH as reflected in its annual meeting is the strong engagement of students; students are actively engaged in poster sessions, presentations, and satellite sessions. Accordingly, we see the Annual Student Manuscript Contest as an excellent opportunity to collaborate with CUGH to help advance the future generation of global health technical excellence and leadership.
